# Adjuvant capecitabine plus oxaliplatin after D2 gastrectomy in Japanese patients with gastric cancer: a phase II study

**DOI:** 10.1007/s10120-016-0606-4

**Published:** 2016-03-08

**Authors:** Nozomu Fuse, Hideaki Bando, Keisho Chin, Seiji Ito, Takaki Yoshikawa, Akira Tsuburaya, Masanori Terashima, Yoshiyuki Kawashima, Tetsu Fukunaga, Masahiro Gotoh, Yasunori Emi, Kazuhiro Yoshida, Eiji Oki, Seiji Takahashi, Hiroshi Kuriki, Kumi Sato, Mitsuru Sasako

**Affiliations:** 1 Department of Gastrointestinal Oncology, National Cancer Center Hospital East, 6-5-1 Kashiwanoha, Kashiwa, Chiba, 277-8577 Japan; 2 Department of Gastroenterology, Cancer Institute Hospital of Japanese Foundation for Cancer Research, Tokyo, Japan; 3 Department of Gastroenterological Surgery, Aichi Cancer Center Hospital, Aichi, Japan; 4 Department of Gastrointestinal Surgery, Kanagawa Cancer Center, Kanagawa, Japan; 5 Division of Gastric Surgery, Shizuoka Cancer Center, Shizuoka, Japan; 6 Division of Gastroenterological Surgery, Saitama Cancer Center, Saitama, Japan; 7 Department of Gastroenterological and General Surgery, St. Marianna University School of Medicine, Kanagawa, Japan; 8 Cancer Chemotherapy Center, Osaka Medical College, Osaka, Japan; 9 Department of Surgery, Saiseikai Fukuoka General Hospital, Fukuoka, Japan; 10 Department of Surgical Oncology, Graduate School of Medicine, Gifu University, Gifu, Japan; 11 Department of Surgery and Science, Kyusyu University Hospital, Fukuoka, Japan; 12 Pharmaceutical Research and Development Department, Yakult Honsha Co., Ltd., Tokyo, Japan; 13 Clinical Science and Strategy Department, Chugai Pharmaceutical Co., Ltd., Tokyo, Japan; 14 Upper GI Division, Department of Surgery, Hyogo College of Medicine, Hyogo, Japan

**Keywords:** Adjuvant treatment, Feasibility study, Gastric cancer, Japanese study, XELOX

## Abstract

**Background:**

Adjuvant chemotherapy with XELOX (capecitabine plus oxaliplatin) has been shown to be beneficial following resection of gastric cancer in South Korean, Chinese, and Taiwanese patients. This phase II study (J-CLASSIC-PII) was undertaken to evaluate the feasibility of XELOX in Japanese patients with resected gastric cancer.

**Methods:**

Patients with stage II or III gastric cancer who underwent curative D2 gastrectomy received adjuvant XELOX (eight 3-week cycles of oral capecitabine, 1000 mg/m^2^ twice daily on days 1–14, plus intravenous oxaliplatin 130 mg/m^2^ on day 1). The primary endpoint was dose intensity. Secondary endpoints were safety, proportion of patients completing treatment, and 1-year disease-free survival (DFS) rate.

**Results:**

One hundred patients were enrolled, 76 of whom completed the study as planned. The mean dose intensity was 67.2 % (95 % CI, 61.9–72.5 %) for capecitabine and 73.4 % (95 % CI, 68.4–78.4 %) for oxaliplatin, which were higher than the predefined age-adjusted threshold values of 63.4 % and 69.4 %, respectively, and the study therefore met its primary endpoint. The 1-year DFS rate was 86 % (95 % CI, 77–91 %). No new safety signals were identified.

**Conclusions:**

The feasibility of adjuvant XELOX in Japanese patients with resected gastric cancer is similar to that observed in South Korean, Chinese, and Taiwanese patients in the Capecitabine and Oxaliplatin Adjuvant Study in Stomach Cancer (CLASSIC) study. Based on findings from this study and the CLASSIC study, the XELOX regimen can be considered an adjuvant treatment option for Japanese gastric cancer patients who have undergone curative resection.

**Electronic supplementary material:**

The online version of this article (doi:10.1007/s10120-016-0606-4) contains supplementary material, which is available to authorized users.

## Introduction

Gastric cancer is prevalent in Asian countries including Japan, South Korea, and China, as well as in South America [1]. The incidence of gastric cancer in Japan was estimated at 29.9 per 100,000 in 2012, giving Japan the third highest incidence in the world [1]. In Japan, 5-year postoperative survival rates of 92 % for stage IA disease, 85 % for stage IB, 70 % for stage II, 47 % for stage IIIA, 29 % for stage IIIB, and 15 % for stage IV disease have been reported [2].

Even after R0 resection, disease recurrence occurs at a constant rate depending on disease stage [2], and adjuvant therapies have been widely investigated for several decades. In Europe, perioperative chemotherapy is considered the standard of care [3], whereas postoperative chemoradiotherapy is a standard of care in the United States (U.S.) based on the results of the landmark INT-0116 study [4, 5]. Significant variations exist throughout the world in the surgery adopted in managing resectable gastric cancer. In the U.S., although D2 dissection is recommended in treatment guidelines [3, 6], common practice has been to perform a D1 lymph node dissection based on the results of phase III studies [7, 8], whereas surgeons in East Asian countries and Europe consider the D2 dissection to be standard practice [3, 9, 10]. In Japan, the Adjuvant Chemotherapy Trial of S-1 for Gastric Cancer (ACTS-GC) study, in which more than 1000 patients with stage II/III gastric cancer after R0 resection were randomized to postoperative S-1 (tegafur, gimeracil, and oteracil) or surgery alone, demonstrated a statistically significant benefit for adjuvant chemotherapy. The hazard ratio (HR) for recurrence-free survival at 3 years was 0.62 [95 % confidence interval (CI), 0.50–0.77] and the HR for death at 5 years was 0.669 (95 % CI, 0.540–0.828) [11, 12]. The capecitabine and oxaliplatin adjuvant study in stomach cancer (CLASSIC) study conducted in South Korea, China, and Taiwan demonstrated a survival benefit for capecitabine plus oxaliplatin (XELOX) in patients with stage II–IIIB gastric cancer following curative D2 gastrectomy, with an HR for disease-free survival (DFS) of 0.56 (95 % CI, 0.44–0.72) for XELOX compared with surgery alone at the 3-year analysis [13] and an HR for death of 0.66 (95 % CI, 0.51–0.85) for XELOX versus surgery alone at the 5-year analysis [14].

Although the XELOX regimen was well tolerated in the CLASSIC study [13, 14], the platinum-based adjuvant chemotherapy regimen, S-1 plus cisplatin, has been reported to be less feasible in Japanese patients unless the first cycle of cisplatin is omitted [15]. Currently, data demonstrating the feasibility of an adjuvant XELOX regimen containing a platinum agent after curative gastrectomy in Japanese patients are not available. We therefore conducted the present phase II study (J-CLASSIC-PII) to evaluate the feasibility of the XELOX regimen in Japanese patients.

## Patients and methods

### Study design

This was a single-arm, phase II study of XELOX in patients with histologically confirmed stage II (excluding those diagnosed pathologically as having T1 or T3N0 disease) or III (excluding those diagnosed pathologically as having T4bN1/N2/N3 disease) gastric adenocarcinoma [Japanese Classification of Gastric Cancer (JCGC), 14th edition] [16]. Patients had no prior treatment for gastric cancer other than surgery, were aged 20 years or older, and had a Karnofsky performance status of 70 % or more. All patients had undergone D2 lymph node dissection with confirmed R0 resection. Patients were enrolled within 6 weeks of surgery for the primary disease, and 4 weeks or more must have elapsed between major surgery and the start of treatment. Adequate hematological function (neutrophil count ≥1.5 × 10^3^/μl, platelet count ≥10.0 × 10^4^/μl, hemoglobin ≥9.0 g/dl), hepatic function [total bilirubin ≤1.5 times the upper limit of normal (ULN), aspartate aminotransferase, alanine aminotransferase, and alkaline phosphatase ≤2.5 × ULN], and renal function (serum creatinine ≤1.5 × ULN and creatinine clearance ≥50 ml/min) were required. The main eligibility criteria of this study were similar to those of the CLASSIC study [13], with the exception of disease staging, which was based on the Japanese classification [16].

Patients were enrolled using a central, interactive web response system. In total, 100 patients were to be enrolled, 50 with stage II disease and 50 with stage III disease. The study was approved by the institutional review board at each participating institution and was performed in accordance with the Declaration of Helsinki and Good Clinical Practice Guidelines. All patients provided written informed consent.

### Treatment

Treatment consisted of capecitabine (1000 mg/m^2^ twice daily on days 1–14 of each cycle) and oxaliplatin (130 mg/m^2^ intravenously on day 1 of each cycle). The first administration began within 1 week of enrollment. Treatment continued for eight 3-week cycles or until discontinuation in response to adverse events, recurrence, or decision by investigator or patient. If oxaliplatin was discontinued as a result of any adverse events, capecitabine monotherapy was allowed. Prophylactic antiemetic therapy for oxaliplatin was administered, for which 5HT_3_ antagonists and dexamethasone were recommended.

Treatment delays, suspensions, or dose modifications were performed as in the CLASSIC study [13]. XELOX therapy with capecitabine and/or oxaliplatin was delayed in the event of grade ≥2 hematological or nonhematological toxicity. Capecitabine was also suspended in the event of any grade ≥3 hematological toxicity and/or any grade ≥2 nonhematological toxicity judged to be capecitabine related and was not resumed until the toxicity had resolved to grade ≤1. After the occurrence of grade ≥3 hematological toxicity or grade ≥2 nonhematological toxicity, the capecitabine dose was reduced to 75 % of the original dose. In the case of a second incidence of grade ≥2 nausea or vomiting, capecitabine dose modifications were made as just described.

After the occurrence of any grade 3 oxaliplatin-related nonhematological toxicity, the oxaliplatin dose was reduced to 100 mg/m^2^ in the subsequent cycle. Oxaliplatin was discontinued in the event of grade ≥4 toxicity.

In case of adverse events judged by the investigator to have a causal relationship to only one of the drugs, dose reduction of the other drug was not necessary. Once reduced, the dose of either drug was not subsequently increased; however, the dose of capecitabine could be increased after discontinuation of oxaliplatin.

Each patient was followed up for 1 year from the start of treatment.

### Assessments

Patients underwent chest, abdominal, and pelvic imaging with magnetic resonance imaging or computed tomography, using a contrast medium if possible, just before starting adjuvant chemotherapy, at 6 months and 1 year after the start of treatment, and when clinically indicated. Evaluation of disease recurrence was performed centrally by an independent efficacy and safety committee.

Adverse events were graded according to the Common Terminology Criteria for Adverse Events (CTCAE) version 4.03.

### Statistical analysis

The primary endpoint of the study was dose intensity, defined as the proportion of the cumulative dose of each drug divided by the total dose of the drug that would have been administered if the patient had received eight cycles without any suspension or dose reduction (planned dose). Based on this definition, the mean dose intensity achieved in the CLASSIC study was 68.2 % for capecitabine and 74.3 % for oxaliplatin. Adjuvant XELOX would be regarded as feasible in Japanese patients if the dose intensities for capecitabine and oxaliplatin in this setting were equivalent to those seen in the CLASSIC study. Point estimates for the dose intensity of each drug were calculated and compared with age-adjusted threshold levels. Threshold levels were calculated as follows: the CLASSIC study population was age-adjusted according to the age distribution observed in the ACTS-GC study [12], which suggested that Japanese patients in the present study would be older than those in the CLASSIC study, and the distribution of the mean dose intensity for the CLASSIC study was calculated by simulation. We considered a threshold to be 2.5 % of the distribution adjusted for age for capecitabine and oxaliplatin, i.e., 63.4 % for capecitabine and 69.4 % for oxaliplatin. A sample size of 100 patients was needed to ensure the mean dose intensity of both agents exceeded the threshold with around 80 % power.

Secondary endpoints were safety, proportion of patients completing treatment (defined as those who completed eight cycles of XELOX or capecitabine alone after discontinuation of oxaliplatin, irrespective of dose reductions, delays, or suspensions), and 1-year DFS rate. DFS was defined as the time from the start of treatment to the time of recurrence of the original gastric cancer, development of a new gastric cancer, or death from any cause.

Time-to-event endpoints were analyzed using Kaplan–Meier survival methods; point estimate of 1-year DFS rate and its 95 % CI were also calculated. DFS in patient subgroups was a pre-specified analysis. Statistical analyses were performed with SAS version 9.2.

The study was registered at JAPIC Clinical Trial Information: JapicCTI-121873.

## Results

### Patient characteristics

Between July 2012 and July 2013, 100 patients were included at 12 institutions in Japan, 41 with stage II disease and 59 with stage III disease. All 100 patients received the treatment and were included in the safety analysis and full analysis sets. Baseline patient characteristics are shown in Table 1.

**Table 1:** Demographics and baseline characteristics (*n* = 100)

Characteristic	*N*
Median age, years (range)	62.0 (29–79)
Mean age, years (±SD)	60.2 (10.6)
Male, *n* (%)	53 (53)
Mean body surface area, m^2^ (±SD)	1.56 (0.16)
Karnofsky performance status, *n* (%)	
100	74 (74)
90	24 (24)
80	2 (2)
JCGC stage^a^, *n* (%)	
IIA	8 (8)
IIB	33 (33)
IIIA	23 (23)
IIIB	16 (16)
IIIC	20 (20)
AJCC stage^b, c^, *n* (%)	
II	50 (50)
IIIA	25 (25)
IIIB	13 (13)
IV	12 (12)
Tumor location, *n* (%)	
Esophagogastric junction	8 (8)
Fundus	15 (15)
Body	25 (25)
Antrum	32 (32)
Fundus + body	3 (3)
Body + antrum	9 (9)
Whole gastric	6 (6)
Other^d^	2 (2)
Nodal category^a^, *n* (%)	
N0	9 (9)
N1	28 (28)
N2	27 (27)
N3a	24 (24)
N3b	12 (12)
Tumor category^a^, *n* (%)	
T2	26 (26)
T3	31 (31)
T4a	43 (43)
Surgical procedure, *n* (%)	
Total gastrectomy	36 (36)
Distal gastrectomy	64 (64)

*AJCC* American Joint Committee on Cancer, *JCGC* Japanese Classification of Gastric Carcinoma, *SD* standard deviation

^a^14th edition

^b^6th edition

^c^Classified N3b ≥ 16 metastatic nodes into stage IV according to the AJCC 6th edition criteria

^d^Multiple primary lesions were included

### Treatment

Patients were treated for a median of eight cycles. A total of 76 patients (76 %) completed the study as planned. Of these, 57 patients completed 8 cycles of XELOX that consisted of both capecitabine and oxaliplatin and 19 patients completed 8 cycles of XELOX, including cycles of capecitabine monotherapy. Twenty-four patients did not complete the treatment because of adverse events (*n* = 11), disease relapse (*n* = 5), refusal by the patient (*n* = 5), or other reasons (*n* = 3). Completion rates were higher in younger (aged <65 years) versus older patients (aged ≥65 years), and in those treated with distal versus total gastrectomies (Table 2).

**Table 2:** Treatment completion in all patients and according to patient subgroups

	No. of patients	Completed treatment, *n* (%)
All patients	100	76 (76)
Age		
<65 years	65	53 (82)
≥65 years	35	23 (66)
Sex		
Male	53	42 (79)
Female	47	34 (72)
JCGC stage^a^		
II	41	30 (73)
III	59	46 (78)
AJCC stage^b^		
II	50	37 (74)
III	38	29 (76)
IV	12	10 (83)
Surgical procedure		
Total gastrectomy	36	23 (64)
Distal gastrectomy	64	53 (83)

*AJCC* American Joint Committee on Cancer, *JCGC* Japanese Classification of Gastric Carcinoma

^a^14th edition

^b^6th edition

The mean dose intensity was 67.2 % (95 % CI, 61.9–72.5 %) for capecitabine and 73.4 % (95 % CI, 68.4–78.4 %) for oxaliplatin (Table 3). These were higher than the predefined age-adjusted threshold values of 63.4 % for capecitabine and 69.4 % for oxaliplatin, and the study therefore met its primary endpoint. Higher dose intensities were achieved in men, patients who underwent distal procedures, and those with more advanced disease. Older patients achieved a slightly lower dose intensity than their younger counterparts.

**Table 3:** Dose intensity in all patients and according to patient subgroups

	No. of patients	Mean dose intensity (95 % CI), %	
		Capecitabine	Oxaliplatin
All patients	100	67.2 (61.9–72.5)	73.4 (68.4–78.4)
Age			
<65 years	65	68.9 (62.5–75.3)	76.1 (70.0–82.1)
≥65 years	35	64.0 (54.3–73.7)	68.5 (59.5–77.4)
Sex			
Male	53	74.1 (67.8–80.4)	78.4 (72.3–84.4)
Female	47	59.3 (50.9–67.8)	67.8 (59.7–75.9)
JCGC stage^a^			
II	41	64.6 (55.6–73.6)	71.6 (63.4–79.8)
III	59	69.0 (62.4–75.6)	74.7 (68.2–81.1)
AJCC stage^b^			
II	50	65.1 (57.0–73.1)	71.1 (63.8–78.5)
III	38	65.4 (56.7–74.1)	73.9 (65.0–82.7)
IV	12	81.6 (72.8–90.4)	81.4 (72.0–90.9)
Surgical procedure			
Total gastrectomy	36	62.3 (52.2–72.5)	68.0 (58.6–77.4)
Distal gastrectomy	64	69.9 (63.8–76.0)	76.5 (70.7–82.3)

*AJCC* American Joint Committee on Cancer, *JCGC* Japanese Classification of Gastric Carcinoma

^a^14th edition

^b^6th edition

### Safety

All patients had at least one adverse event. Adverse events occurring in ≥10 % of patients are summarized in Table 4. The most common all-grade, any-cause adverse events were peripheral sensory neuropathy (*n* = 94), nausea (*n* = 87), neutropenia (*n* = 76), diarrhea (*n* = 67), and decreased appetite (*n* = 66). Grade ≥3 events occurred in 71 patients (71 %), the most common of which were neutropenia (*n* = 33), decreased appetite (*n* = 17), peripheral sensory neuropathy (*n* = 14), and nausea (*n* = 10). Twenty serious adverse events occurred in 16 patients; 11 patients had treatment-related serious adverse events, including vomiting (*n* = 2), decreased appetite (*n* = 2), and nausea, lower gastrointestinal hemorrhage, drug-induced liver injury, memory impairment, malaise, bacteremia, orthostatic hypotension, and drug hypersensitivity (*n* = 1 each). One patient died as a result of superior mesenteric artery thrombosis (related to treatment).

**Table 4:** Adverse events occurring in more than 10 % of patients (*n* = 100)

Adverse event, *n* (%)	All grades	Grade 3/4
Peripheral sensory neuropathy	94 (94)	14 (14)
Nausea	87 (87)	10 (10)
Neutropenia	76 (76)	33 (33)
Diarrhea	67 (67)	2 (2)
Decreased appetite	66 (66)	17 (17)
Hand–foot syndrome	48 (48)	0
Vomiting	46 (46)	5 (5)
Infusion site pain	45 (45)	1 (1)
Thrombocytopenia	43 (43)	6 (6)
Fatigue	43 (43)	6 (6)
Leukopenia	32 (32)	1 (1)
Malaise	28 (28)	2 (2)
Dysgeusia	28 (28)	0
Stomatitis	26 (26)	0
Constipation	25 (25)	0
Hepatic function abnormal	21 (21)	0
Weight decreased	20 (20)	1 (1)
Nasopharyngitis	14 (14)	0
Pigmentation disorder	12 (12)	0
Edema, peripheral	12 (12)	0
Drug hypersensitivity	11 (11)	1 (1)

Adverse events profiles according to age and surgical procedure are shown in Tables 5 and 6, respectively. The incidences of any-grade peripheral sensory neuropathy, nausea, neutropenia, diarrhea, and decreased appetite in younger and older patients were 92 % versus 97 %, 88 % versus 86 %, 75 % versus 77 %, 68 % versus 66 %, and 66 % versus 66 %, respectively. The incidences of any-grade peripheral sensory neuropathy, nausea, neutropenia, diarrhea, and decreased appetite in patients with total gastrectomy and with distal gastrectomy were 92 % versus 95 %, 97 % versus 81 %, 67 % versus 81 %, 69 % versus 66 %, and 69 % versus 64 %, respectively.

**Table 5:** Adverse events occurring in more than 20 % of patients according to age

Adverse event, *n* (%)	Younger than 65 years (*n* = 65)		Older than or equal to 65 years (*n* = 35)	
	All grades	Grade 3/4	All grades	Grade 3/4
Peripheral sensory neuropathy	60 (92)	9 (14)	34 (97)	5 (14)
Nausea	57 (88)	5 (8)	30 (86)	5 (14)
Neutropenia	49 (75)	20 (31)	27 (77)	13 (37)
Diarrhea	44 (68)	1 (2)	23 (66)	1 (3)
Decreased appetite	43 (66)	10 (15)	23 (66)	7 (20)
Hand–foot syndrome	31 (48)	0	17 (49)	0
Vomiting	34 (52)	3 (5)	12 (34)	2 (6)
Infusion site pain	31 (48)	1 (2)	14 (40)	0
Thrombocytopenia	30 (46)	3 (5)	13 (37)	3 (9)
Fatigue	26 (40)	2 (3)	17 (49)	4 (11)
Leukopenia	22 (34)	0	10 (29)	1 (3)
Malaise	22 (34)	2 (3)	6 (17)	0
Dysgeusia	21 (32)	0	7 (20)	0
Stomatitis	16 (25)	0	10 (29)	0
Constipation	19 (29)	0	6 (17)	0
Hepatic function abnormal	14 (22)	0	7 (20)	0
Weight decreased	12 (19)	1 (2)	8 (23)	0
Edema peripheral	5 (8)	0	7 (20)	0

**Table 6:** Adverse events occurring in more than 20 % of patients according to surgical procedure

Adverse event, *n* (%)	Total gastrectomy (*n* = 36)		Distal gastrectomy (*n* = 64)	
	All grades	Grade 3/4	All grades	Grade 3/4
Peripheral sensory neuropathy	33 (92)	6 (17)	61 (95)	8 (13)
Nausea	35 (97)	6 (17)	52 (81)	4 (6)
Neutropenia	24 (67)	12 (33)	52 (81)	21 (33)
Diarrhea	25 (69)	1 (3)	42 (66)	1 (2)
Decreased appetite	25 (69)	6 (17)	41 (64)	11 (17)
Hand–foot syndrome	16 (44)	0	32 (50)	0
Vomiting	15 (42)	3 (8)	31 (48)	2 (3)
Infusion site pain	15 (42)	0	30 (47)	1 (2)
Fatigue	17 (47)	3 (8)	26 (41)	3 (5)
Thrombocytopenia	10 (28)	1 (3)	33 (52)	5 (8)
Leukopenia	10 (28)	1 (3)	22 (34)	0
Dysgeusia	9 (25)	0	19 (30)	0
Malaise	7 (19)	1 (3)	21 (33)	1 (2)
Stomatitis	9 (25)	0	17 (27)	0
Constipation	9 (25)	0	16 (25)	0
Weight decreased	9 (25)	1 (3)	11 (17)	0
Hepatic function abnormal	5 (14)	0	16 (25)	0

The dose used in the XELOX regimen (either capecitabine or oxaliplatin) was modified in 92 patients, primarily as a result of neutropenia (*n* = 70). Both the XELOX components were discontinued as a result of an adverse event in 11 patients (neutropenia, *n* = 7; increased gamma glutamyltransferase, *n* = 2; other reasons *n* = 5); oxaliplatin alone was discontinued in 20 patients (peripheral sensory neuropathy, *n* = 10; drug hypersensitivity, *n* = 5; neutropenia, *n* = 2; other reasons, *n* = 3). The capecitabine dose was reduced in 78 patients, the most common reasons being neutropenia (*n* = 23), decreased appetite (*n* = 20), diarrhea (*n* = 16), fatigue (*n* = 10), and hand–foot syndrome (*n* = 9). Oxaliplatin dose reductions were required in 66 patients, primarily as a result of neutropenia (*n* = 24), decreased appetite (*n* = 13), and peripheral sensory neuropathy (*n* = 13). XELOX cycle delays occurred in 82 patients, the most common reasons for which were neutropenia (*n* = 68), thrombocytopenia (*n* = 23), peripheral sensory neuropathy (*n* = 10), hand–foot syndrome (*n* = 7), and decreased appetite (*n* = 6).

### Efficacy

Overall, the 1-year DFS rate was 86 % (95 % CI, 77–91 %). DFS is summarized in Supplementary Fig. 1. According to JCGC, 14th edition, the 1-year DFS rates for 41 patients with stage II and for 59 patients with stage III were 87 % (95 % CI, 72–94 %) and 84 % (95 % CI, 71–92 %), respectively. Recurrences were reported in 12 patients: in the peritoneum and/or ascites (*n* = 7), liver (*n* = 3), lung or pleural effusion (*n* = 2), and lymph nodes (*n* = 2). No locoregional recurrences were observed.

## Discussion

The dose intensity achieved in Japanese patients exceeded the threshold pre-specified by age adjustment of the dose intensity in the CLASSIC study, and this study therefore achieved its primary endpoint. The age distribution in this study was similar to the ACTS-GC study as expected. We can therefore regard adjuvant XELOX to be feasible in Japanese patients with gastric cancer.

Treatment completion rates observed in the present study were similar to those achieved in the CLASSIC study, with 76 % of patients meeting the study definition of treatment completion compared with 67 % in the CLASSIC study. Administration of six or more cycles of XELOX was considered important for the efficacy of adjuvant chemotherapy in the CLASSIC study [14], and we believed it would be important to continue treatment for a sufficient period in our study, with appropriate dose modifications and careful management of adverse events. Indeed, dose reduction or suspension did not appear to negatively impact on efficacy in the CLASSIC study [14]; completing treatment as scheduled, even with dose modification, appeared to be beneficial for patients. The fact that similar dose intensity and treatment completion rates were achieved in Japanese patients as in CLASSIC, and that the time from surgery to enrollment was the same in both studies, suggests that the results of the CLASSIC study can be extrapolated to Japanese patients.

Overall, the safety profile of XELOX in the Japanese patients in this study was comparable with the safety of XELOX in the South Korean, Chinese, and Taiwanese patients in the CLASSIC study. There were no clear differences in observed adverse events, except the incidences of some events. The incidence of all-grade peripheral sensory neuropathy was higher in the present study (94 % vs. 10 % in the CLASSIC study), as were the incidences of hand–foot syndrome (48 % vs. 19 %), and nausea (87 % vs. 66 %). In terms of grade 3/4 events, the incidences of neutropenia (33 % vs. 22 %), peripheral sensory neuropathy (14 % vs. <1 %), and decreased appetite (17 % vs. 5 %) were also higher in this study versus CLASSIC. Even taking into consideration that Bang et al. reported all-grade and grade 3/4 peripheral neuropathy, as distinct from peripheral sensory neuropathy, in 56 % and 2 % in the CLASSIC study [13], the overall incidence of neuropathy was higher in Japanese patients. The incidences of neuropathy and hand–foot syndrome in the present study were similar to those observed in Japanese patients with metastatic colorectal cancer [17]. We recommend that the adverse events more frequently observed in Japanese patients be monitored and managed carefully to ensure completion of the scheduled XELOX therapy.

The higher incidence of some adverse events in Japanese patients compared with CLASSIC could not be explained by surgical procedure and age distribution. We had assumed that the incidences of adverse events would increase with older age; we observed, however, that although fatigue occurred more frequently in older patients, no other differences in adverse events were observed between older and younger patients in this study (Table 5). Another possibility was that some adverse events would be more common in our study than in CLASSIC as a result of the greater number of patients with upper lesions (esophagaogastric junction, fundus, fundus and body, and whole gastric tumors) in this study compared with CLASSIC (32 % vs. 15 %, respectively). Patients with upper lesions are more likely to have undergone total gastrectomy, which might result in an increase in adverse events; in fact, although the incidence of nausea was slightly higher in patients who had undergone a total gastrectomy, no apparent differences were observed in adverse events between patients with total and distal gastrectomy (Table 6). Further investigation is required to clarify the reasons for the differences in adverse event profiles in the two studies.

The results of the present study were also comparable with those of the ACTS-GC study, which was performed in Japanese patients [11]. The periods between surgery and the start of treatment and the treatment completion rates were similar in both the present study and the S-1 arm of the ACTS-GC study. With regard to tolerability, the XELOX regimen had a different adverse event profile to the S-1 regimen in the ACTS-GC study, although available data for the ACTS-GC study are limited. In the XELOX regimen, peripheral sensory neuropathy, nausea, neutropenia, diarrhea, and decreased appetite were observed in more than 50 % of the patients, and the incidences of nausea, vomiting, and thrombocytopenia were more than 10 % higher than in the S-1 regimen. On the other hand, anemia, anorexia, diarrhea, leukopenia, and fatigue were observed in more than 50 % of the patients in the S-1 regimen, whereas the incidences of leukopenia, fatigue, and pigmentation were more than 10 % higher with S-1 than with the XELOX regimen.

The recommended duration of adjuvant XELOX therapy for gastric cancer is 6 months, which is shorter than the duration of the current standard treatment in Japan, i.e., 1 year of S-1. This shorter treatment period might be beneficial for some patients, even though the XELOX regimen requires intravenous administration in a hospital and the adverse events associated with the two regimens are different. One other potential difference between the two regimens is cost: the total cost of XELOX is higher than that of S-1 monotherapy [18].

This study had some limitations, as it was a single-arm study performed in a limited number of patients. Additionally, long-term survival data [i.e., DFS and overall survival] were not obtained. However, we assumed that achieving the similar dose intensity and completion rate for XELOX as in CLASSIC would result in efficacy similar to that observed in CLASSIC.

In conclusion, the present study has shown that the safety and feasibility of adjuvant XELOX in Japanese patients with resected gastric cancer are equivalent to those observed in South Korean, Chinese, and Taiwanese patients in the CLASSIC study. Based on the results of this study and CLASSIC, the XELOX regimen can be considered an appropriate adjuvant treatment option for Japanese patients with gastric cancer who have undergone curative resection.

## Electronic supplementary material

Below is the link to the electronic supplementary material.

Supplementary material 1 (PDF 462 kb)
