# Supplementary material for: Adjuvant capecitabine plus oxaliplatin after D2 gastrectomy in Japanese patients with gastric cancer: a phase II study
Source: Gastric Cancer. 2016 Mar 8;20(2):332–40. doi: 10.1007/s10120-016-0606-4 (PMC5321693; doi:10.1007/s10120-016-0606-4)

Supplementary Figure 1. Disease-free survival in Japanese patients with resected gastric cancer treated with adjuvant XELOX

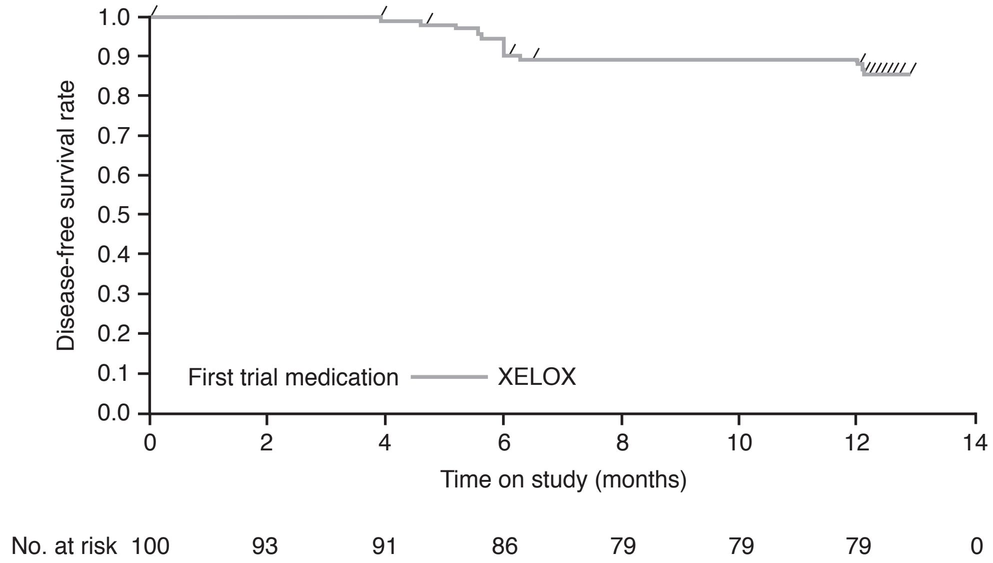

Supplement: Supplementary file 1 — Supplementary material 1 (PDF 462 kb) [file 10120_2016_606_MOESM1_ESM.pdf]
